# Inflammatory Myofibroblastic Tumor: A Rarely Seen Submucosal Lesion of the Stomach

**DOI:** 10.1155/2013/328108

**Published:** 2013-03-19

**Authors:** Deniz Arslan, Şeyda Gündüz, Deniz Tural, Mükremin Uysal, Ali Murat Tatlı, Cumhur İbrahim Başsorgun, Gülsüm Özlem Elpek, Hasan Şenol Coşkun, Hakan Şat Bozcuk, Burhan Savaş

**Affiliations:** ^1^Division of Medical Oncology, Department of Internal Medicine, Medical Faculty, Akdeniz University, 07058 Antalya, Turkey; ^2^Division of Medical Oncology, Department of Internal Medicine, Cerrahpasa Medical Faculty, Istanbul University, 34098 Istanbul, Turkey; ^3^Department of Pathology, Medical Faculty, Akdeniz University, 07058 Antalya, Turkey

## Abstract

Inflammatory myofibroblastic tumor (IMT) is a rare mesenchymal benign tumor which is generally seen in children and in young adults. It is especially located in the lungs. In histopathological examination, neoplastic fusiform cells originating from a subtype of accessory immune system cells which are called fibroblastic reticulum cells are seen (Kouichi and Youichirou, 2008). Although IMT is histopathologically benign, imaging methods show its tendency for local recurrence and invasion. In most of the cases, it may not be possible to make a distinction whether it is malign or benign. Complete surgical resection is the most important treatment method. In this study, we have discussed the findings of our case having a gastric submucosal located IMT in light of the current literatures.

## 1. Introduction

Inflammatory myofibroblastic tumor (IMT) is a rarely seen mesenchymal benign tumor which is generally seen in children and in young adults [1]. It especially inhibits the lungs [2]. In histopathological examination, neoplastic fusiform cells originating from a subtype of accessory immune system cells which are called fibroblastic reticulum cells are seen [3]. Although IMT is benign histopathologically, it is a disease which is in tendency of local recurrence and invasion by imaging methods. In most of the cases, it may not be possible to make a distinction whether it is malign or benign [4]. Despite various treatment approaches, the best method is complete surgical resection. If complete surgical resection cannot be applied, symptomatic treatment should be considered, and more aggressively chemotherapy and radiotherapy should be considered if there is a disease progression or a progression in the symptoms [5].

In this study, we have discussed the findings of our case having a gastric submucosal located IMT in light of the current literatures.

## 2. Case Presentation

**Figure 1:**
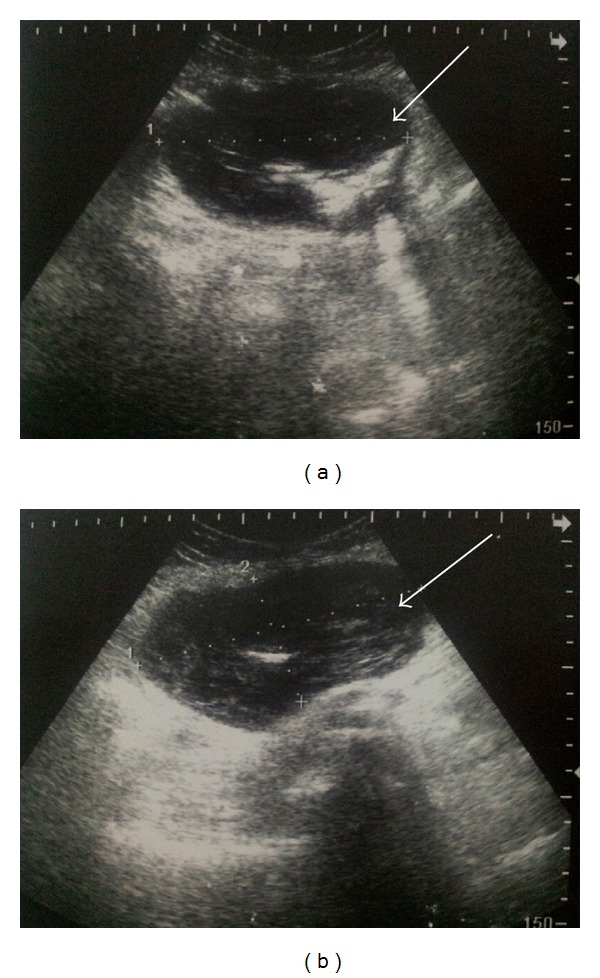
Abdomen USG. 114 × 52 × 99 mm sized hypoechogenic mass lesion in front of the main vascular structures in the abdomen midline in USG with calcification focuses inside (marked with an arrow).

**Figure 2:**
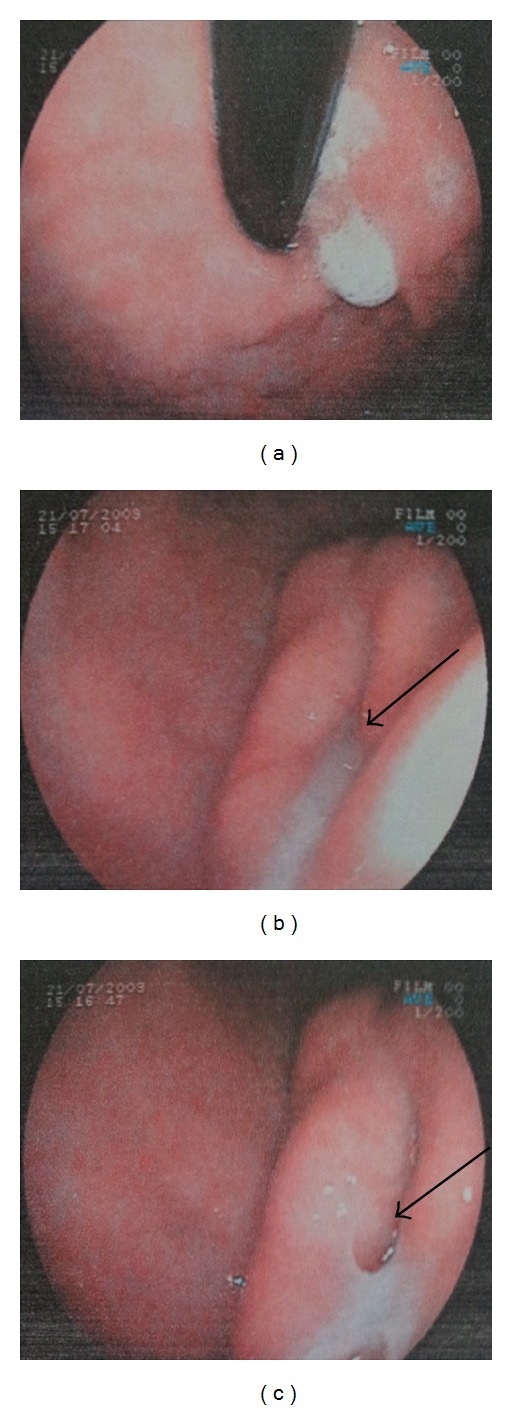
Upper GIS endoscopy. A mass in the antrum and a fistula orifice with purulent flux were determined (marked with an arrow).

**Figure 3:**
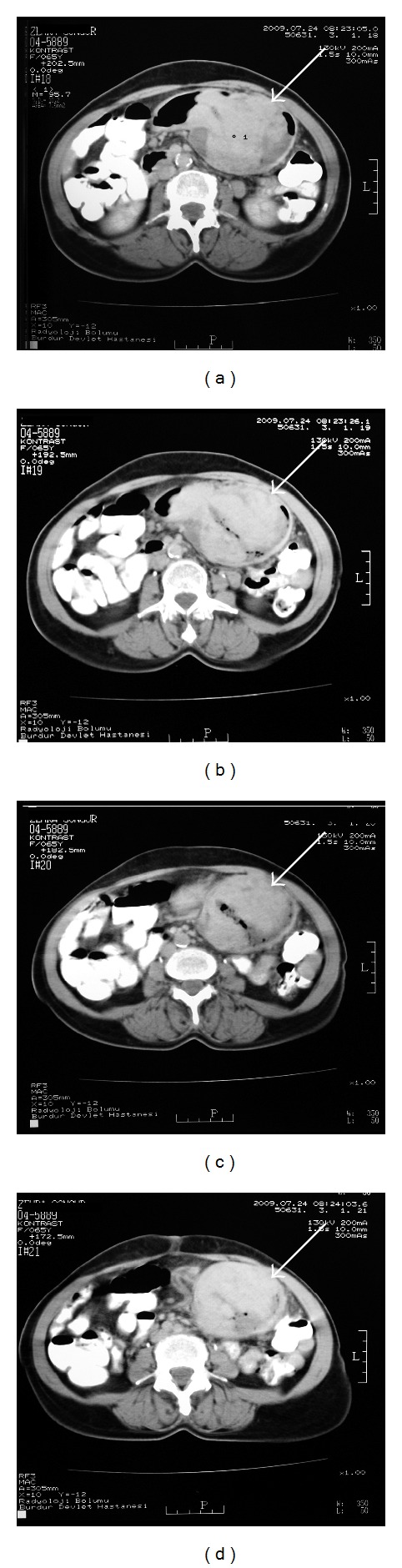
Abdomen computerized tomography (CT). In the right side of the abdomen midline, over the transverse column, a heterogeneous hyperdense solid mass with the size of 7,5 × 10 × 11 cm having cystic areas and air densities inside was determined (marked with an arrow).

**Figure 4:**
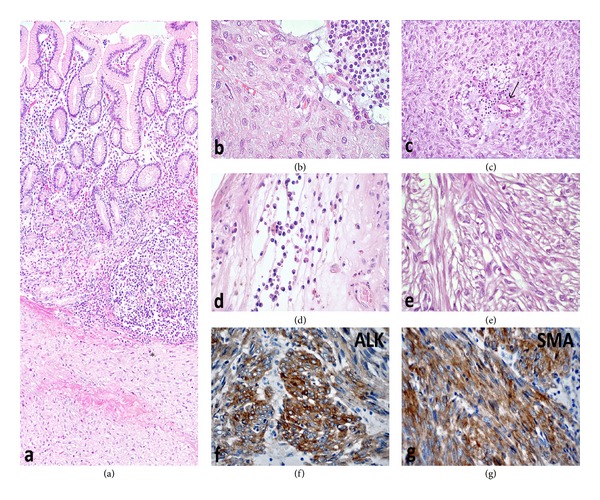
Microscopy of IMT. (a) A cellular tumor (asterisk) infiltrating into submucosa and mucosa of the stomach (hematoxylin and eosin stain H & E, ×50). (b) Alternating lymphoplasmacytic infiltration and tumor cells (H & E, ×200). (c) In some areas, scattered inflammatory cells and many vascular structures (arrow) are seen (H & E, ×100). (d) Plasma cells forming clusters in a myxoid background (H & E, ×100). (e) Spindle-shaped myofibroblastic tumor cells show a fascicular arrangement with relatively few inflammatory cells (H & E, ×400). (f) Strong cytoplasmic staining for anaplastic lymphoma kinase (ALK) in tumor cells (Anti-ALK antibody, counterstained with hematoxylin, ×200). (g) In some areas, tumor cells also express smooth muscle actin (SMA) (Anti-SMA antibody, counterstained with hematoxylin, ×200).

A sixty-five-year-old female patient was admitted to the hospital with dyspepsia, intermittent pain in the epigastrium complaints for approximately 3 years and loss of appetite, nausea, vomiting, and weight loss (5 kg) complaints for the last 1 month. In USG, 114 × 52 × 99 mm sized well-circumscribed hypoechogenic mass lesion with calcifications inside was observed in front of the main vascular structures in the midline of the abdomen (Figure 1). In the upper gastrointestinal system (UGS) endoscopy, a mass in the antrum and a fistula orifice with purulent flux were determined (Figure 2). In the computerized tomography (CT), in the right side of the abdomen midline, over the transverse column, a heterogeneous hyperdense solid mass with the size of 7,5 × 10 × 11 cm having cystic areas and air densities inside was determined (Figure 3). Via surgical stomach wedge resection, solid and cystic mass lesion was removed by dissecting from the transverse column meso. The cellular pathology of resected mass which had a repulsive growth into the gastric mucosa was reported as pseudocapsulated, myofibroblastic, ill-circumscribed, bulky spindle-shaped nucleus located in the loose strome and myxoid with eosinophilic cytoplasm lymphoid follicle formations and plasma cell groups mainly more intense in tumor periphery. SMA, S-100, Demsin, Ki 67, p53, and CD 117 stains were applied to the incisions immunohistochemically (IHC), and staining was only observed by SMA (Figure 4). The patient has been followed up in remission for 37 months after the complete surgical resection.

## 3. Discussion

IMT is a rarely seen tumor which is also known as inflammatory pseudotumor. While it is generally asymptomatic, nonspecific symptoms such as fever, malaise, weight loss, and abdominal pain may be seen [6, 7]. Although it is benign, development of malign transformation, metastasis, or recurrence is also reported [2, 6–8]. It is typically seen in preschoolers and young adults, and it usually affects women (woman/man, 4 : 1) [2, 6]. Its etiology is not exactly known. It is thought to be arisen as a result of a period after an infection, trauma, biliary obstruction, and a surgical operation, and it is histopathologically formed of inflammatory cells (plasma cells, lymphocytes, macrophages, eosinophils, and neutrophils), fibroblasts, and various collagen deposits [6, 9, 10]. It is also considered to be related to lymphoma since ALK-TPM3 fusion is seen similar to large cell anaplastic lymphoma [5]. It mostly affects the lungs; however, it may also affect multiple organs such as the liver, pancreas, spleen, lymph node, breast, kidney, bladder, orbita, and central nervous system [1, 2]. IMT of the stomach is uncommon, and because it may be confused with gastrointestinal stromal tumor (GIST) from the other submucosal lesions, the definitive diagnosis should be verified by an IHC study [11]. When there is stomach involvement, the lesion is 3–10 cm (average 7 cm) sized, and it tends to be located especially in cardia, antrum, and in the prepyloric region. It frequently infiltrates the adjacent organs such as the esophagus, duodenum, pancreas, spleen, liver, ligament, peritoneal cavity, and large omentum [2]. Sometimes it is difficult to differentiate the benign and malign mass lesions by radiological methods [4]. Presurgical USG and CT may help to determine the extragastric invasion [2].

Radiologically, there is no finding specific to the inflammatory myofibroblastic tumor [12]. In abdominal radiology, intestine segments may be relocated due to the calcifications inside the tumor and the mass effect [4]. In USG and CT exams, it may be seen as solid and sometimes heterogeneous and lobular, well-limited, and calcified nodular lesions. Additional to such findings, because it has an infiltrative appearance, it may not be differentiated from many of the metastatic diseases [2, 4]. Significant vascular signals may be received in Doppler US [13].

The insufficiency of clinical symptoms in IMT mandates the diagnosis to be made via biopsy. Sometimes it can show spontaneous regression; thus, conservative treatment and followup are recommended. When no response is given to the treatment, surgical resection is applied [3, 6–8]. Complete surgical resection is the most efficient treatment method. If incomplete resection is applied to the pseudotumor, generally a local recurrence is seen within a year [2]. When it is impossible to apply resection, an incomplete resection may be applied, or in case of a severe morbidity, radiotherapy (RT) and chemotherapy (cyclosporine, methotrexate, azathioprine, and cyclophosphamide) may be applied. High dose steroid treatment may be given in orbital pseudotumors [5, 8].

As a result, IMT should be kept in mind for a gastric submucosally located mass lesion with a purulent flux fistula in an old aged woman. The success of the treatment is high in the patients when complete surgical resection is applied as a primary treatment.
